# Differential pH-dependent cellular uptake pathways among foamy viruses elucidated using dual-colored fluorescent particles

**DOI:** 10.1186/1742-4690-9-71

**Published:** 2012-08-30

**Authors:** Kristin Stirnnagel, Dorothee Schupp, Aurélie Dupont, Volodymyr Kudryavtsev, Juliane Reh, Erik Müllers, Don C Lamb, Dirk Lindemann

**Affiliations:** 1 Institute of Virology, Medizinische Fakultät "Carl Gustav Carus", Technische Universität Dresden, Fetscherstr. 74, 01307 Dresden, Germany; 2 CRTD / DFG-Center for Regenerative Therapies Dresden - Cluster of Excellence, Technische Universität Dresden, Dresden, Germany; 3 Department of Chemistry, Center for NanoScience (CeNS) and Center for Integrated Protein Science, Munich (CIPSM), Ludwig-Maximilians-Universität, Munich, Germany; 4 Department of Physics, University of Illinois at Urbana-Champaign, Urbana, IL, USA

**Keywords:** Retrovirus, Foamy virus, Entry, Disassembly, Intracellular targeting, Time-lapse microscopy, Live-cell imaging

## Abstract

**Background:**

It is thought that foamy viruses (FVs) enter host cells via endocytosis because all FV glycoproteins examined display pH-dependent fusion activities. Only the prototype FV (PFV) glycoprotein has also significant fusion activity at neutral pH, suggesting that its uptake mechanism may deviate from other FVs. To gain new insights into the uptake processes of FV in individual live host cells, we developed fluorescently labeled infectious FVs.

**Results:**

N-terminal tagging of the FV envelope leader peptide domain with a fluorescent protein resulted in efficient incorporation of the fluorescently labeled glycoprotein into secreted virions without interfering with their infectivity. Double-tagged viruses consisting of an eGFP-tagged PFV capsid (Gag-eGFP) and mCherry-tagged Env (Ch-Env) from either PFV or macaque simian FV (SFVmac) were observed during early stages of the infection pathway. PFV Env, but not SFVmac Env, containing particles induced strong syncytia formation on target cells. Both virus types showed trafficking of double-tagged virions towards the cell center. Upon fusion and subsequent capsid release into the cytosol, accumulation of naked capsid proteins was observed within four hours in the perinuclear region, presumably representing the centrosomes. Interestingly, virions harboring fusion-defective glycoproteins still promoted virus attachment and uptake, but failed to show syncytia formation and perinuclear capsid accumulation. Biochemical and initial imaging analysis indicated that productive fusion events occur predominantly within 4–6 h after virus attachment. Non-fused or non-fusogenic viruses are rapidly cleared from the cells by putative lysosomal degradation. Quantitative monitoring of the fraction of individual viruses containing both Env and capsid signals as a function of time demonstrated that PFV virions fused within the first few minutes, whereas fusion of SFVmac virions was less pronounced and observed over the entire 90 minutes measured.

**Conclusions:**

The characterized double-labeled FVs described here provide new mechanistic insights into FV early entry steps, demonstrating that productive viral fusion occurs early after target cell attachment and uptake. The analysis highlights apparent differences in the uptake pathways of individual FV species. Furthermore, the infectious double-labeled FVs promise to provide important tools for future detailed analyses on individual FV fusion events in real time using advanced imaging techniques.

## Background

As virus replication is strictly dependent on infecting susceptible cells, viruses have evolved several strategies to enter their host. Within the host, their journey of amplification is initiated by adsorption to specific cellular receptor molecules on the target cell surface. Often, two kinds of binding events are involved. In a first attachment step, viruses are concentrated at the cell surface. This process is relatively nonspecific, frequently involving cell surface carbohydrate structures and surfing along cellular protrusions such as filopodia [1,2]. Following this first step, a second, more specific interaction with the specific cellular receptor of proteinaceous, lipid or carbohydrate nature promotes viral entry. Depending on the virus species, different cellular uptake pathways are exploited (reviewed in [3,4]). Membrane-enveloped viruses can penetrate host cells by either viral glycoprotein-mediated fusion at the plasma membrane or inducing endocytic uptake (reviewed in [5-7]). Viral capsids released by fusion at the plasma membrane have to break through the actin matrix [8]. Subsequent to this internalization process, free capsids are further transported towards the cell center along microtubules by hijacking cellular motor proteins like dynein or dynactin [3,9,10]. In contrast, endocytosed viruses are challenged to release their capsid into the cytosol before the endosomal content is delivered to lysosomes, where degradation occurs. To overcome this endosomal trap, some viruses take advantage of the pH conditions inside endosomes. The low pH of mature or late endosomes can trigger the fusion activity of viral glycoproteins, subsequently activating capsid release by merging the viral and the endosomal membranes [6].

Whereas the uptake pathways of some viruses are well-defined, we are only at the beginning of understanding how foamy viruses (FVs), a special type of complex retroviruses, infect host cells. Like all members of the *Retroviridae*, FVs integrate their genome into the host cell chromosomes. Besides this classical feature of retroviruses, other replication steps used by FVs are distinct from orthoretroviruses (e.g. HIV-1), but bear homology to hepadnaviruses. Therefore, FVs are classified into a separate subfamily, the *Spumaretrovirinae*[11]. FVs are characterized by an extremely broad host-range. The nature of their ubiquitous receptor, which seems to be evolutionarily conserved, has not yet been conclusively determined [12]. Recently, involvement of proteoglycans and heparan sulfate as attachment receptors for FVs was reported as they greatly enhance target cell susceptibility towards these viruses [13-15]. Previously, we had reported that all glycoproteins of a variety of FV species display a pH-dependent fusion activity peaking around pH 5.5, when examined using a cell-cell fusion assay [16]. However, one species, the prototype FV (PFV), also displayed significant fusion activity at neutral pH. The pH-dependent fusion activity of all analyzed FV Env proteins and the sensitivity to lysosomotropic agents (e.g. Bafilomycin A1) suggest an endocytic entry mechanism of FVs [16]. Additional support for this assumption comes from early reports showing endosomal SFVmac (macaque simian FV) uptake in infected cells by using electron microscopy [17].

Like other viruses, FVs can also hijack the cellular cytoskeleton for intracellular trafficking of incoming viral capsids. This piggyback transport along microtubules is thought to be achieved by direct interaction of the PFV Gag protein with the light chain 8 (LC8) of the dynein motor protein complex [18] after the fusion process. Furthermore, it was also reported that intact naked PFV capsids accumulate at the MTOC, which presumably disassemble later on at the centrosomes [19-21]. These observations led to the assumption that PFV capsid-envelope separation already occurs upon the route of viral particles to the centrosomes. Currently, it is not known whether the FV glycoprotein mediated fusion happens at the plasma membrane or after endocytosis.

To gain insights into the uptake processes leading to release of the capsid into the cytosol, we generated infectious double-tagged FV particles composed of eGFP-tagged PFV capsids and mCherry-labeled envelope proteins of PFV or SFVmac. The uptake and trafficking processes of these two types of fluorescent FV particles upon target cell entry were analyzed by biochemical methods in bulk populations as well as time-lapsed wide-field and confocal imaging analysis in individual living cells.

## Results/Discussion

### PFV uptake

**Figure 1:**
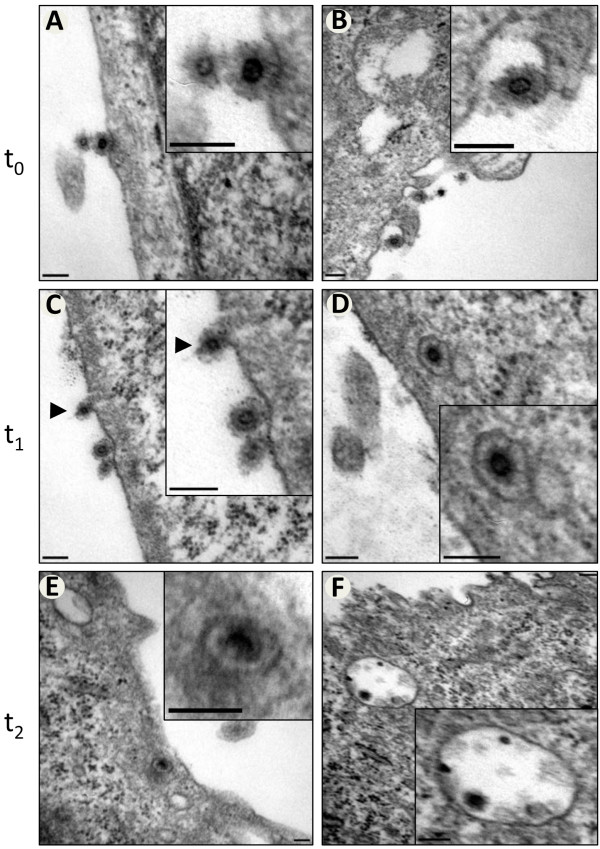
**Representative electron micrographs showing the involvement of endocytic pathways in PFV uptake.** HeLa cells were incubated with untagged, wildtype PFV particles at ~10°C. After 30 minutes incubation, the cells were (**A-B**) either fixed (t_0_) or (**C-F**) warmed to 37°C for an additional 10 minutes (t_1_) or 30 minutes (t_2_) prior to fixation. Scale bars: 100 nm

The fusion activity of all FV glycoproteins is pH-dependent with higher activity at low pH [16]. Therefore, it is thought that FV uptake follows endocytic routes. As a proof-of-principle, we aimed to analyze the early uptake processes of PFV infected cells using electron microscopy. HeLa cells were incubated with wild-type PFV particles at low temperatures to avoid virus uptake and increase loading of the cellular plasma membrane with bound viruses. Following incubation and intensive washing, these cells were either fixed directly (t_0_) or warmed to 37°C for 10 minutes (t_1_) or 30 minutes (t_2_) prior to fixation to allow entry of viral particles. Ultrathin sections of these samples were analyzed by electron microscopy. At the earliest time point (t_0_), PFV particles were found to be bound to the extracellular site of the plasma membrane, sometimes associated with cellular protrusions (Figure 1A, B). After 10 minutes incubation at 37°C, the internalization of some viruses was observed while many were still attached to the cell surface (Figure 1C, D). The majority of viruses that entered the cell were enclosed by a lipid membrane, which we assume to be the result of endocytic uptake. For example, the virus enlarged in Figure 1D is in close proximity to a putative virus-empty endosome/vesicle. In a few cases, viruses apparently fusing at the plasma membrane were observed (Figure 1C, particle marked with arrowhead). Thirty minutes after infection, the majority of viruses were localized inside the cell either in smaller endosomes or bigger putative multivesicular bodies (Figure 1E, F).

### Development and characterization of single and double-labeled FV particles

**Figure 2:**
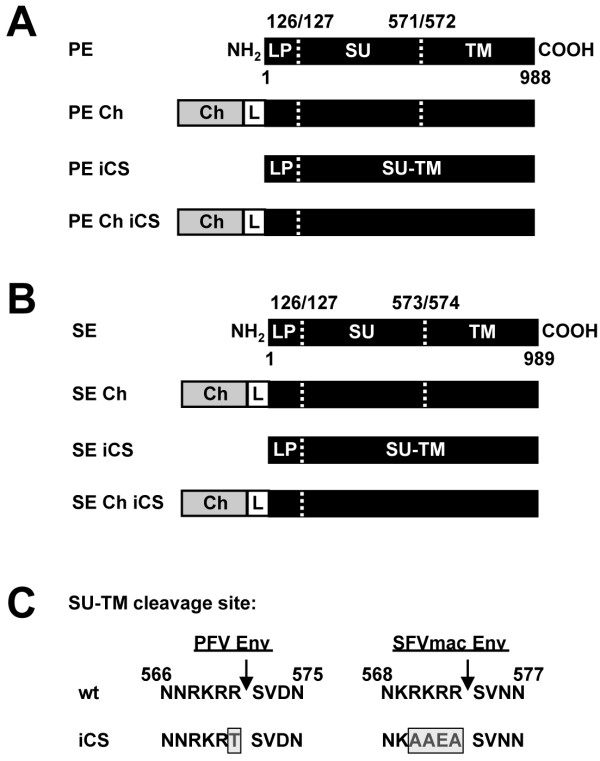
**Schematic outline of FV mCherry-Env fusion proteins and Env cleavage site mutants.** The mCherry tag (Ch) with a flexible glycine-serine-linker (L) was fused to the N-terminus of the *env* open reading frames of (**A**) PFV or (**B**) SFVmac. (**C**) Inactivation of the surface-transmembrane cleavage site (SU/TM) was achieved by introducing a single point mutation (R_571_T) in the PE/PE Ch sequence or exchanging four amino acids (RKRR_570-573_AAEA) in the SE/SE Ch sequence. These mutations resulted in PE iCS and SE iCS or the mCherry-Env fusion constructs of PE Ch iCS and SE Ch iCS. The cleavage sites in the Env proteins are indicated as dashed lines. Abbreviations: LP, leader peptide; PE, PFV Env; SE, SFVmac Env; Ch, mCherry; iCS, inactivated cleavage site

**Figure 3:**
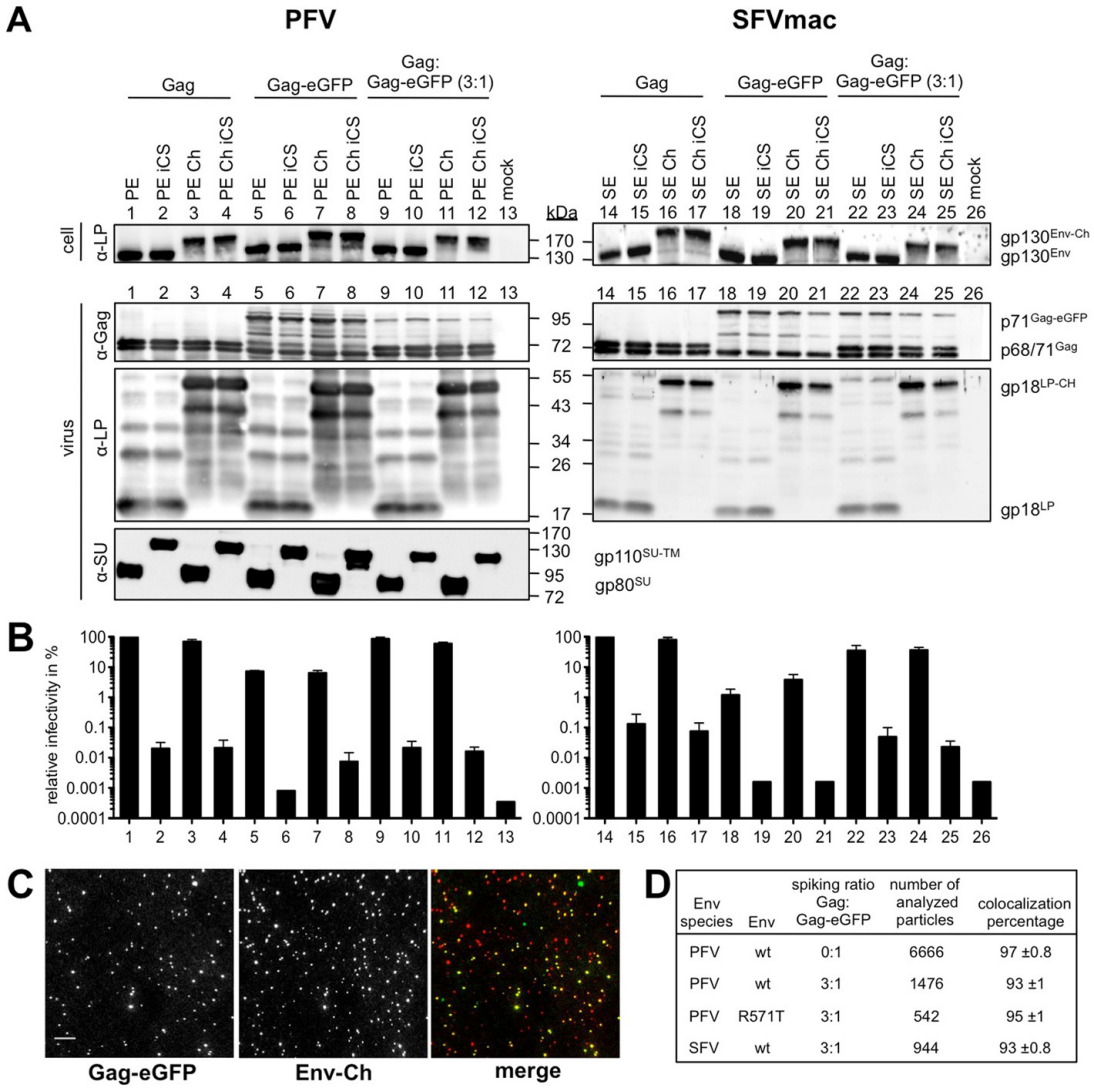
**Characterization of single and double-tagged FV particles.** (**A**) Cellular and particle-associated protein expression analysis of either PFV or SFVmac Env constructs. Representative immunoblots of 293T cell lysates (cell) and purified viral particles (virus). For PFV particles, 293T cells were cotransfected with puc2MD9, pcoPP and (1–4) pcoPG4, (5–8) pcoPG4 CeGFP, (9–12) pcoPG4 : pcoPG4 CeGFP (3:1) as well as (1, 5, 9) pcoPE, (2, 6, 10) pcoPE iCS, (3, 7, 11) pcoPE Ch, (4, 8, 12) pcoPE Ch iCS. For the production of SFVmac Env pseudotyped particles, 293T cells were cotransfected with puc2MD9, pcziPol and (14–17) pcziGag4, (18–21) pcziGag4 CeGFP, (22–25) pcziGag4 : pcziGag4 CeGFP (3:1) as well as (14, 18, 22) pciSE, (15, 19, 23) pciSE iCS, (16, 20, 24) pciSE Ch, (17, 21, 25) pciSE Ch iCS. As a control, cells were only transfected with pUC19 (13, 26). The viral proteins were detected using antibodies specific for PFV Gag (α-Gag), PFV or SFVmac Env LP (α-LP) and PFV Env SU (α-SU). (**B**) Transduction efficiency shown as relative infectivity of cell culture supernatants containing PFV (bar 1–13) or SFVmac (bar 14–26) Env harboring foamy viral particles. The obtained relative values of one representative experiment are shown. The sample with untagged Gag and Env was arbitrarily set to 100%. (**C**) 2D colocalization analysis of spotted FV particles. Exemplary wide-field images were obtained with purified Gag-eGFP and Ch-Env (PE Ch) tagged particles. Scale bar 10 μm. (**D**) Summary of the obtained colocalization percentages of the indicated viruses

To gain more insights into the uptake, transport and fate of incoming viruses, we developed dual-labeled FVs for use in fluorescence live-cell microscopy. Previously, we characterized PFV particles with fluorescent protein (FP)-tagged capsids [13]. Here, we report on the generation of FP-tagged FV envelope proteins used to establish double-tagged viruses. MCherry (Ch)-Env fusion proteins were generated with fusion competent wild-type envelope proteins of PFV Env (PE) or SFVmac Env (SE) (PE Ch, SE Ch) (Figure 2). Fusion incompetent variants of these tagged FV glycoproteins were also generated by amino acid exchanges (R_571_T or RKRR_570-573_AAEA), leading to inactivation of the Env surface-transmembrane (SU-TM) subunit furin cleavage site (PE Ch iCS and SE Ch iCS) (Figure 2). Untagged, single or double-tagged FV particles were generated using a replication-deficient 4-component vector system (Gag, Env, Pol, and genomic RNA). Using this system, variants of PFV or SFVmac Env expression vectors were either cotransfected with packaging vectors encoding untagged PFV Gag, Gag-eGFP or Gag:Gag-eGFP at a ratio of 3:1 as indicated in Figure 3. Cellular expression levels (cell) as well as particle-associated protein compositions (virus) were analyzed by immunoblotting (Figure 3A). All Ch-Env fusion proteins (160 kDa) were produced at equal expression levels compared to the untagged envelope protein gp130^Env^. The biosynthesis of the FV glycoprotein is rather unusual. The mature leader peptide (LP) subunit (here N-terminally tagged with mCherry) is generated by post-translational processing of furin or furin-like protease, has a type II membrane topology, and is a physical constituent of the glycoprotein complex in released virions [22]. In general, introduction of FPs into FV particles neither influenced the protein composition of the particles nor the particle release efficiencies. The levels of incorporated Pol proteins were indistinguishable for untagged, single or double-tagged particles (data not shown). In Ch-Env containing particles, the major form of the wild-type leader peptide (LP, gp18^LP^) was shifted up to about 50 kDa (Figure 3A, lane 1–12, 14–25). A second prominent Ch-LP-derived protein of about 40 kDa was also detected, which presumably arises from an internal mCherry cleavage reported to be necessary for chromophore maturation of mCherry [23-26]. Besides the LP sera, SU-specific antibodies raised against PFV Env were used to prove the inactivation of the SU-TM cleavage site in the iCS variants of PFV Env. Due to inactivation of the furin cleavage site between SU and TM domains, the iCS Env precursor protein is just cleaved once between the LP and SU domain. Therefore, the SU and TM domains stay together resulting in a PFV SU-TM protein of about 110 kDa. Only the PFV iCS mutant showed this SU-TM protein and a lack of wild-type gp80^SU^ (Figure 3A, lane 2, 4, 6, 8, 10, 12). A similar biochemical characterization of processing of the different SFVmac Env proteins was not possible due to the lack of a SFVmac SU-specific antibody.

The fusion of Ch to PFV or SFVmac Env only marginally influenced the relative infectivity of extracellular viruses (Figure 3B, bar 1, 3, 14, 16). In contrast, comparison of infectivities of particles harboring the authentic mCherry-tagged wild type PFV Env (Figure 3B, bar 1, 3) to those containing the respective fusion-defective PFV Env (iCS) variants (Figure 3B, bar 2, 4) revealed a 5,000-fold difference. Similarly a 1,000-fold difference was observed for the corresponding wild type (Figure 3B, bar 14, 16) and fusion-defective (Figure 3B, bar 15, 17) glycoproteins of SFVmac.

Double-tagged particles composed of Gag-eGFP and Ch-Env showed a 10 to 100-fold reduction of viral titers (Figure 3B, bar 1, 7 PFV; bar 14, 20 SFVmac). This is in accordance with previous reports that showed that different types of retroviral particles composed of only Gag-eGFP had a similarly decreased infectivity [13,27]. We also could confirm our previous data [13], showing that cotransfection of untagged Gag:Gag-eGFP can rescue the infectivity defect up to almost wild-type levels in the case of PFV Env (Figure 3B, bar 9, 11) and up to 40% using SFVmac Env (Figure 3B, bar 22, 24). Thus, viral functions of double-tagged particles, and the infectivity in particular, are predominantly influenced by the modification of the Gag protein.

Next, we determined the fraction of double-labeled FV particles with respect to the total number of Gag-eGFP particles using fluorescence microscopy. Purified viral particles were allowed to settle on coverslips, and the fluorescent intensities of individual particles in the green channel (Gag-eGFP) and red channel (Ch-Env) were measured (Figure 3C). The percentage of Gag-eGFP signals colocalizing with Ch-Env was calculated for all double-labeled preparations used (Figure 3D). The fraction of double-tagged viruses (with respect to all particles having a Gag-eGFP signal) was found to be between 93 and 97% for all preparations. This is not surprising as the cognate Env protein is required for FV budding [28]. In contrast, only about 50% of all red-labeled particles contained Gag-eGFP (Figure 3C). Attaching a fluorescent protein to the N-terminus of a FV glycoprotein strongly increased the release of capsidless subviral particles [29] that are characterized by Ch-Env only signals (data not shown).

Our approach relies on the fact that FV glycoproteins can be labeled with FP without affecting the functionality of the Env protein. The use of FP-labeled glycoproteins is rare (e.g. [2,30,31]) as Env-FPs are typically non-functional. Other approaches for the generation of double-labeled retroviruses using FP-tags have been reported in the past. For example, HIV-1 has been double-labeled by employing membrane-targeting signal-tagged FP proteins or FP-tagged Vpr co-packaged into HIV-1 particles [32,33]. However, the FV Env-FP system developed in this study holds great promise for the further elucidation of the kinetics and dynamics of processes in the life cycle of retroviruses.

### Differential cell-cell fusion characteristics of various FV Env containing virions

We used the double-labeled FVs to gain insights into viral entry and trafficking in infected cells. The entry pathway of foamy virus particles containing different Env proteins (PFV, SFVmac) in living cells was followed over 24 h using time-lapsed wide-field microscopy.

**Figure 4:**
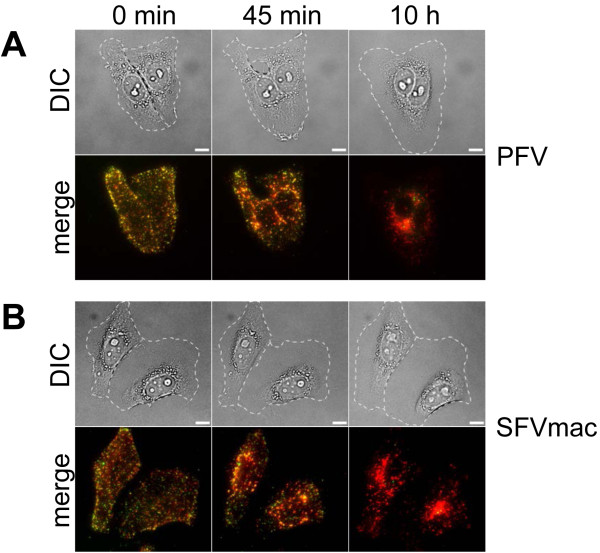
**Cell-cell fusion capacities of PFV and SFVmac Env containing virions.** (**A**) Cells incubated with PFV showed cell-cell fusion of neighboring HeLa cells upon virus loading onto the cell surface, whereas (**B**) exposure to SFVmac failed to mediate plasma membrane fusion of neighboring cells. The upper panels show differential interference contrast (DIC) images of the cells. The lower panels show *z*-projections of fluorescence wide-field images of HeLa cells exposed to double-tagged FV particles where eGFP and mCherry channels have been merged. Z-stacks with 13 planes were taken every 15 minutes. Images are shown from the beginning of the experiment (0 min), after 45 minutes and after 10 hours. See Additional file 1

One major difference between PFV and SFVmac Env containing particles, noticed immediately in the first experiments, was the formation of syncytia from neighboring cells by PFV Env (PFV) harboring virions, but not by SFVmac Env (SFV) containing ones (Figure 4, see Additional file 1). The syncytia formation by PFV Env containing particles was induced very rapidly by a “fusion from without mechanism” of two neighboring cells after virus loading and warming to 37°C (Figure 4A). In contrast, SFVmac Env harboring viruses did not induce the membrane fusion of two neighboring cells, although they were in close contact with each other (Figure 4B). The rate of PFV induced cell-cell-fusion was dependent on the density of growing cells. In the case of higher cell numbers, syncytia with 6 to 10 nuclei could be observed (see Additional file 1).

These observations point to a restriction of SFVmac fusion events to intracellular organelles. Fusion from mature and late endosomes would be consistent with the pH-dependent fusion properties of SFVmac Env described previously [16]. In line with this, Bafilomycin A1 blocked SFVmac Env-mediated transduction of HeLa cells more efficiently than PFV Env-mediated transduction (see Additional file 2). As the PFV glycoprotein displays significantly higher fusion activity at neutral pH, we propose that PFV possesses a fusion-active state already at the plasma membrane where it then can induce syncytia formation [16,34]. Since Chloroquine treatment, an inhibitor of lysosome acidification [35,36], did not dramatically decrease FV infectivity, we assume that fusion from lysosomes does not play an important role in FV entry (see Additional file 2). The analysis of other drugs interfering with the function of cellular kinases (Genistein, [37]), dynamin (Dynasore, [38]) or drugs leading to cholesterol depletion (Nystatin, [39]) did not reveal strong infectivity-decreasing effects as was observed for Bafilomycin A1 (see Additional file 2). With these compounds, the infectivity was either slightly reduced (Chloroquine, Dynasore, Nystatin) or remained essentially unchanged (Genistein). For comparison, VSV-G enveloped HIV pseudoparticles were measured under identical conditions. The differential effects of all drugs on the VSV-G- and the FV Env-mediated infectivity suggest that FV entry triggers a different virus entry pathway than that of the VSV-G envelope.

### Time-lapsed wide-field imaging of FV uptake in individual host cells

**Figure 5:**
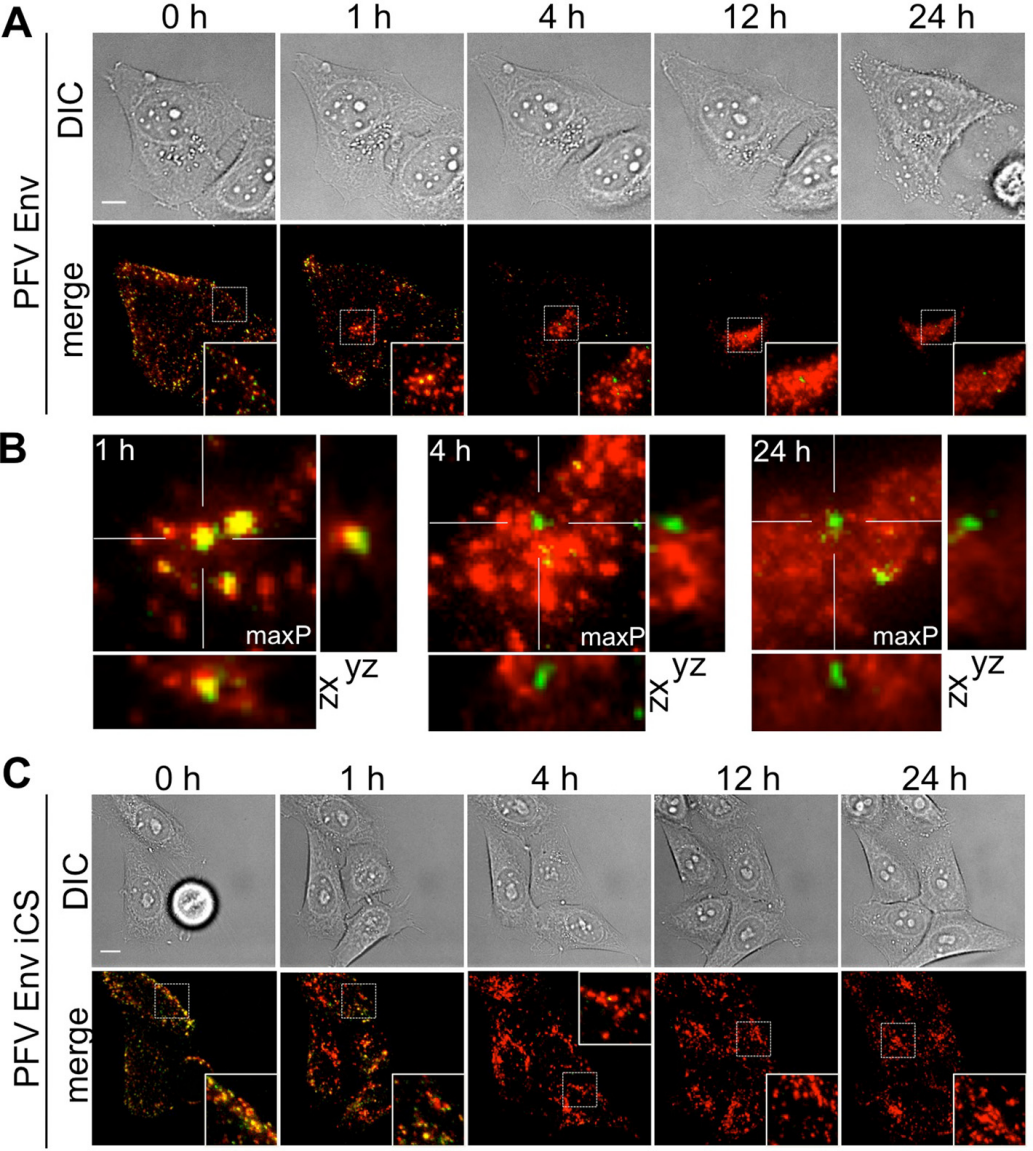
**Time-lapsed imaging of PFV uptake.** HeLa cells were infected with double-tagged viruses consisting of PFV Gag-eGFP and (**A, B**) PE Ch or (**C**) PE Ch iCS. Panel A and C display images from differential interference contrast measurements (DIC) as well as an overlay of the eGFP and mCherry channels (merge) for the selected time points of 0 h, 1 h, 4 h, 12 h and 24 h post virus binding. The fluorescence images are maximum intensity z-projections of z-stacks of 10–12 planes collected using live-cell wide-field microscopy. The scale bar represents 10 μm. Panel B shows an expanded region along with orthogonal views of the perinuclear area from images in panel A at 1 h, 4h and 24 h post virus binding. maxP, maximum intensity projection. See Additional file 3 and Additional file 5

**Figure 6:**
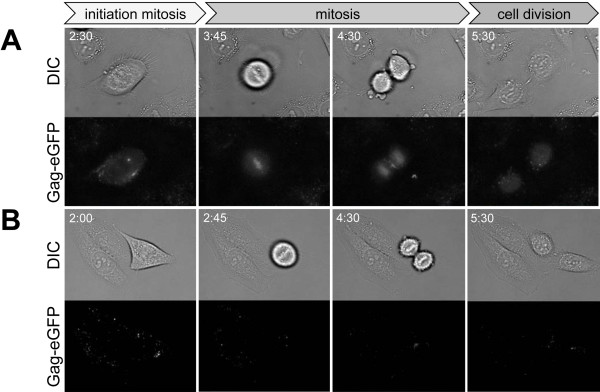
**Gag tethering to host cell chromatin during mitosis.** HeLa cells were infected with double-tagged viruses consisting of PFV Gag-eGFP and (**A**) PE Ch or (**B**) PE Ch iCS. Images from differential interference contrast measurements (DIC) and the eGFP (Gag-eGFP) channels are shown for selected time points (h pb). Z-stacks with 12 planes were collected every 15 minutes. The fluorescence images are maximum intensity z-projections of z-stacks collected using live-cell wide-field microscopy. See Additional file 4

In contrast to the different cell-cell fusion characteristics of PFV Env and SFVmac Env containing particles, the entry route into host cells taken by these two viruses shows similarities (Figure 5 and data not shown). Figure 5A shows the results for fusion-competent PE Ch viruses. At the earliest time point, viruses were bound to the plasma membrane of the cells (Figure 5A, 0 h pb, hours post binding, and Additional file 3). Within the first two hours, almost all wild-type double-labeled viruses entered the cells, yielding an accumulation of signal in the perinuclear region (Figure 5A, B, 1 h pb). After 2–4 h, separate capsid and envelope signals were detected in the majority of cells (75%, n = 32) (Figure 5A, B, 4 h). Capsid aggregates were typically observed as punctate structures close to the nucleus, and the envelope mCherry signal was vesicularly distributed throughout the cell, but started to concentrate at the perinuclear site. This indicates that fusion resulting in capsid and envelope separation had already occurred. In cells undergoing mitosis within the observation period, Gag-eGFP tethered to chromatin was readily observable (Figure 6 A, see Additional file 4). As recently published, this step may represent one pathway for Gag and/or the preintegration complex to gain access to the nucleus and further mediates Gag-tethering to the chromosomes [40,41].

Next, we examined the potential uptake and fate of fusion-incompetent PFV Env (Figure 5C and Additional file 5) or SFVmac Env viral particles. The target cell attachment and initial uptake routes of both types of non-fusogenic viruses were quite similar to the corresponding wild-type viruses. This indicates that the Env fusion activity is not essential for FV attachment and early uptake into host cells and suggests that endocytic uptake pathways are exploited. Within the first hour after exposure of HeLa cells to double-labeled fusion-incompetent PFV particles (PE Ch iCS), we observed accumulation of viruses inside the cell in a similar manner to that of wild-type viruses (Figure 5C, 1 h). However, at 2–4 h pb, almost no separate Gag-eGFP signal was observable inside cells incubated with iCS virus and none of the cells (0%, n = 17) examined in detail showed aggregates of naked capsids at the MTOC, represented as punctate structures close to the nucleus (compare Figure 5C, 4 h to Figure 5A, 4 h). Furthermore, no tethering of Gag-eGFP to chromatin in mitotic cells was detectable (Figure 6B, see Additional file 4). This points to a rapid degradation of non-fusogenic viruses that are unable to release their capsid into the cytoplasm. Therefore, we assume that the productive fusion events occur predominantly within the first 4 h pb and particles that do not succeed in undergoing fusion within this time period are prone for rapid degradation.

In summary, the fusion-incompetent viruses were characterized by three major differences in comparison to the respective wild-type viruses. First, in the case of PFV Env, cells incubated with iCS virions never showed formation of cell-cell fusion (data not shown). Second, no capsid aggregates (Gag-eGFP only signals) were observed in cells exposed to both types of fusion incompetent viruses and the presence of individual capsids was rare (Figure 5C, 4 h, 12 h, 24 h). Third, Gag did not associate with the host cell chromosomes upon cell division (Figure 6B, see Additional file 4).

### Incoming FV capsids accumulate at the MTOC

**Figure 7:**
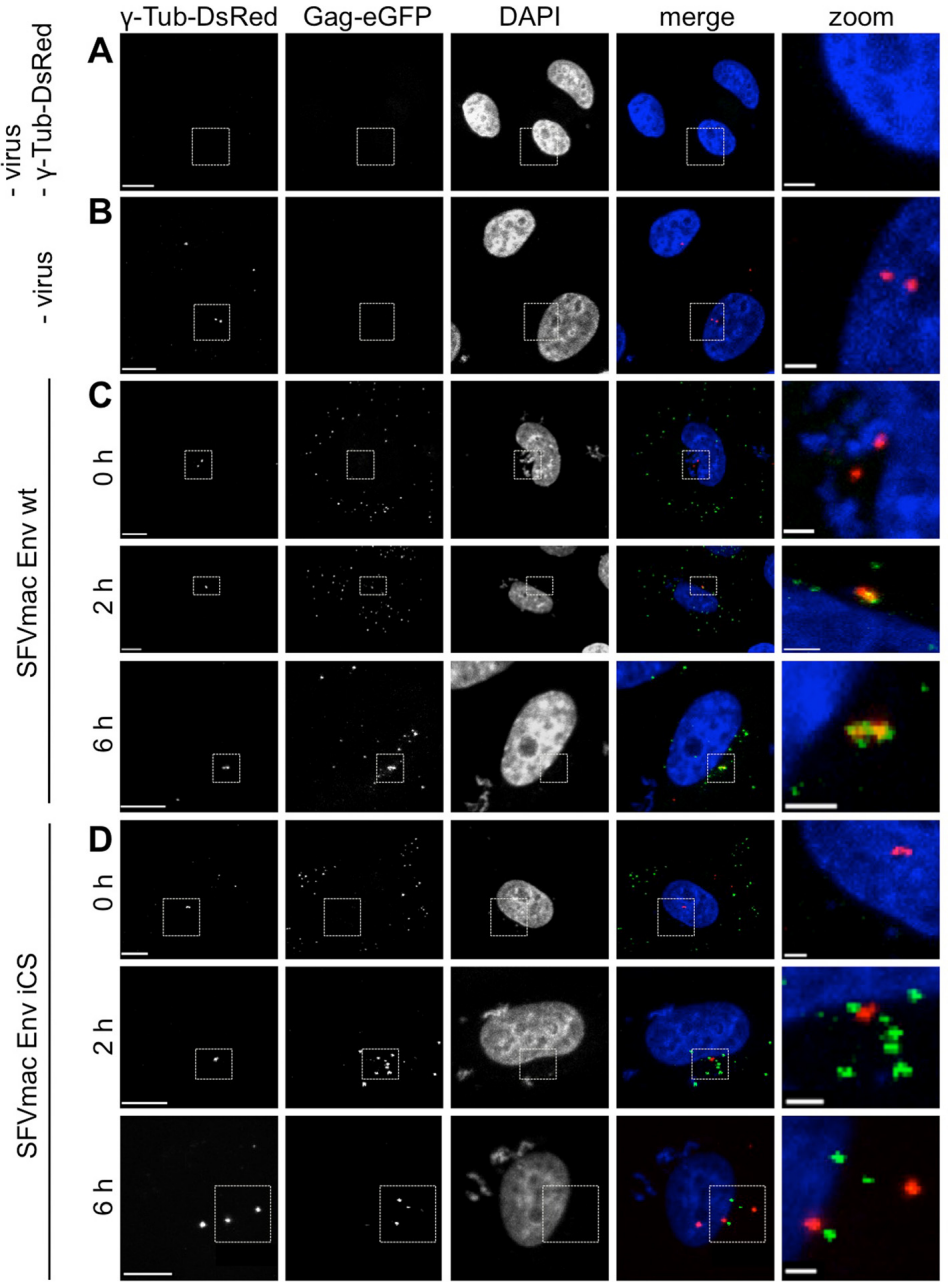
**Colocalization of incoming FV capsids with the MTOC.** PgTubulin-DsRed transfected HeLa cells were incubated with PFV Gag-eGFP tagged SFVmac Env viruses (SE, SE iCS). The samples were fixed with 3% PFA after 0, 2 or 6 h post virus binding at 37°C, and confocal fluorescence images were collected. Representative images for (**A**) control cells, (**B**) pgTubulin-DsRed expressing cells, (**C-D**) pgTubulin-DsRed transfected and (**C**) SFVmac Env wt or (**D**) SFVmac Env iCS incubated cells at 0, 2, 6 h post virus binding are shown. Scale bar: 5 μm

In cells incubated with wild type virus, separate capsid signals accumulated as punctate structures at a perinuclear region (Figure 5A). Depending on the target cell cycle status, the Gag-eGFP capsid staining was detected as a single dot (Figure 5A, 12 h) or doublet (Figure 5A, 24 h). Previous reports [18,20] suggested that this perinuclear region represents the MTOC targeted by incoming capsids. A colocalization analysis of single-labeled Gag-eGFP particles containing SFVmac Env, taken up in cells transfected with dsRed-tagged γ-tubulin, verified that the capsid signals indeed accumulate at the MTOC as shown in Figure 7. In contrast to wild-type viruses, the capsids from the corresponding non-fusogenic iCS viruses accumulated near the γ-tubulin structures of the MTOC but were not tightly associated with the MTOC (Figure 7 C, D). Combining this observation with the wide-field microscopy results of double-labeled viruses presented above strongly suggests that capsids from the non-fusogenic iCS viruses are still enveloped viruses within endosomes, which were trafficked to the cell center.

### Degradation of FV

**Figure 8:**
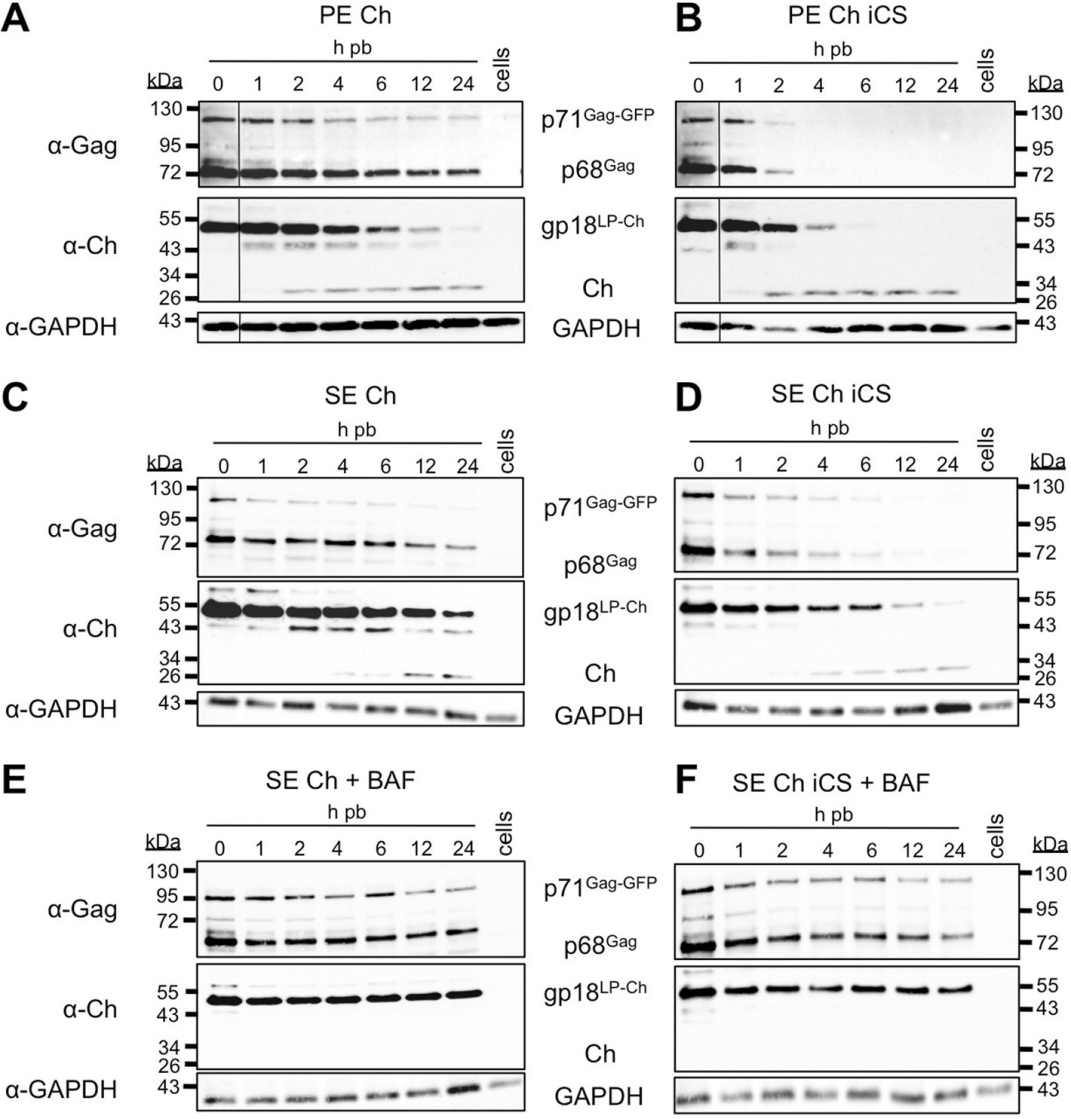
**Analysis of viral protein degradation in infected cells.** (**A-D**) Untreated or (**E, F**) Bafilomycin A1-preincubated HeLa cells were incubated for 30 minutes at ~10°C with double-tagged viruses composed of Gag-eGFP labeled capsids and (**A**) PE Ch, (**B**) PE Ch iCS, (**C, E**) SE Ch, (**D, F**) SE Ch iCS. Subsequently, cells were either lysed immediately (0 h pb) or warmed to 37°C to allow virus entry for the time periods indicated (1–24 h pb). At the respective time points, the cells were lysed, and the cell lysates were analyzed by immunoblotting. The capsid proteins were detected using a PFV Gag-specific antibody (α-Gag) and the LP-Ch protein using a mCherry-specific antibody (α-Ch). Abbreviations: h pb, hours post binding; GAPDH, Glyceraldehyde 3-phosphate dehydrogenase

Unlike the Gag-eGFP signal, both the wild-type and the non-fusogenic viruses surprisingly showed a similar distribution of the Ch-Env signal in the measured cells over time (Figure 5). For both virus types, the Ch-Env fluorescence signal was detected over 24 h pb with signals showing strong accumulation at the perinuclear area (Figure 5). Particularly for the non-fusogenic iCS viruses, which are unable to escape from the endocytic compartment, this was very unexpected. Degradation of iCS virions in lysosomes would be expected to result in a disappearance of eGFP and mCherry signals at a similar rate. To investigate why the entry of non-fusogenic PE Ch iCS viruses should lead to degradation of the tagged capsids but not of the tagged Env-LP, we used biochemical methods to analyze the presence of these two viral proteins in cell lysates of infected cells. HeLa cells were loaded with fusion-competent or fusion-deficient double-labeled viruses (PE Ch, PE Ch iCS, SE Ch, SE Ch iCS) at ~10°C, rinsed, warmed to 37°C, lysed at certain time points and analyzed by immunoblotting (Figure 8A-D). In cells infected with fusion-competent viruses, we observed that the amount of Gag-eGFP and Ch-LP proteins decreased over time, but were still present in cells 24 h pb (Figure 8A, C). In contrast, none of the viral proteins could be detected at 24 h pb in cells exposed to fusion-deficient viruses (Figure 8B, D). The depletion of Gag proteins in cell lysates of fusion-deficient virus infected cells was first observed at 2 h pb. Within 6 h pb, almost all Gag proteins were degraded, which correlates with the lack of eGFP signals observed during live-cell imaging at this time point (Figure 5C). In contrast to the live-cell imaging measurements of mCherry signals, the Ch-LP protein was degraded over time until 4–6 h pb in the cell lysates of PE Ch iCS and until 12–24 h pb in SE Ch iCS exposed cells (Figure 8B, D). However, an additional protein with a molecular weight of about 29 kDa was detected with mCherry-specific antibodies and appeared in the cell lysates of both fusion-competent and fusion-deficient virus exposed cells starting at 2–4 h pb (Figure 8A-D). This suggests that, at later time points during wide-field live cell imaging, the mCherry signals are mainly derived from degradation-resistant mCherry alone and not the Ch-LP fusion protein. For an unknown reason, the mCherry-tag appears to survive lysosomal degradation to a certain extent whereas viral proteins are efficiently cleared. This appears to be a general characteristic of mCherry since survival of the mCherry-tag was also observed when fluorescent protein tags where swapped between Gag and Env proteins (data not shown).

These results also indicate that the fusogenic entry pathway of FV occurs predominantly within the first 4–6 h pb. Viruses that were unable to undergo Env-mediated fusion with cellular membranes within this time period are prone to degradation.

### Influence of Bafilomycin on the viral entry pathway

To further test whether SFVmac Env mediated entry, contrary to PFV Env, is restricted to endocytic uptake, we analyzed the affect of Bafilomycin A1 (BAF) on viral entry. BAF is an inhibitor of endosomal pH acidification [42] and would artificially retain SFVmac Env containing viruses in endosomes by preventing fusion on the one hand and viral degradation on the other hand. The fate of viral proteins of wild type or fusion-deficient SFVmac Env containing particles was examined biochemically upon infection of target cells treated with BAF. In these cell lysates, both Gag-eGFP and Ch-LP were detected by immunoblot until 24 h pb (Figure 8E, F). Furthermore, the occurrence of free mCherry proteins was neither observed in SE Ch nor SE Ch iCS viruses, indicating that the presence of free mCherry in BAF-untreated cells is observed concomitantly with degradation of the incoming Ch-LP of the virus.

Thus, inhibiting endosomal acidification and protein degradation through BAF incubation of cells results in retaining SE Ch particles in endosomal compartments, underlining the involvement of endocytosis in FV uptake.

If Env-mediate fusion is triggered by the lower pH value in endosomes, artificial elevation of the endosomal pH by BAF-treatment should also prevent escape of capsids into the cytoplasm and thus no aggregation of naked capsids at the MTOC in target cells should be observable. This was examined by time-lapsed wide-field imaging analysis of different FVs (PE Ch, SE Ch, SE Ch iCS) in BAF-treated target cells over 12 h (see Additional file 6). All three types of viruses were taken up into the cells, accumulated in the perinuclear region and dual-colored signals were observed until 12 h pb (compare Additional file 6 to Additional file 3, Additional file 5 and Figure 5). In BAF-treated cells incubated with SE Ch or SE Ch iCS particles, no accumulation of naked capsids at the MTOC was detectable. In contrast, cells incubated with PE Ch particles in the presence of BAF still showed the characteristic Gag-GFP punctate structures that represent centrosomal accumulations of naked capsids. Taken together, these results confirm the strictly pH-dependent fusion process of SFVmac Env containing particles after endocytic uptake. In addition, they demonstrate that PFV Env containing particles escape the inhibitory effect of BAF-treatment to a large extent, probably due to viral fusion at neutral pH.

### Confocal time-lapsed analysis of colocalized capsid and Env signal in live cells

In order to get a general and more quantitative impression about the level of fusion activity of the different FV species and the time scale of viral entry and fusion, we evaluated the fraction of enveloped capsids in individual cells over time. Experiments were performed in live cells where the uptake of viruses could be followed in the same subset of cells, thereby decreasing one factor of variability within the measurement. We developed a software program to perform a global 3D colocalization analysis suitable for the data from the live-cell experiments. This program is based on the detection of signals in the Gag-eGFP channel and the presence of a corresponding signal in the opposite Ch-Env channel. In order to avoid artifacts arising from autofluorescence or channel crosstalk, 3D stacks of live-cell images were acquired with alternating excitation [43,44] using a spinning-disk confocal microscope. Low particle numbers per cell were used to avoid random colocalization that would lead to false positive events (see Additional file 7). We also incorporated the full 3D image information obtained from the z-stack in the analysis. For more detailed information, see material and methods. From the analysis, the total number of particles in each channel was obtained as well as the number of colocalizing dual-color particles.

**Figure 9:**
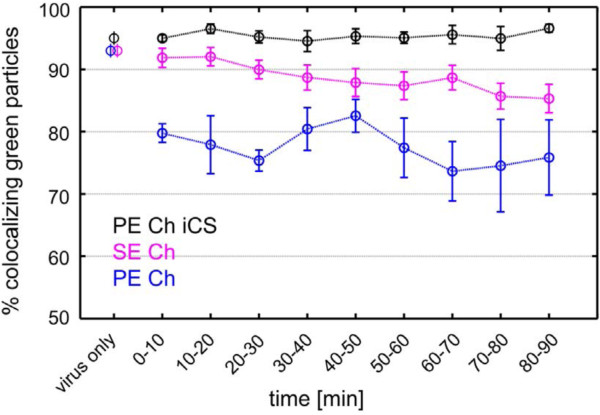
**Loss of Gag-Env colocalization in FVs over time in live cells.** HeLa cells were incubated with double-tagged viruses composed of Gag:Gag-eGFP labeled capsids (3:1) and PE Ch (PE Ch, blue), PE Ch iCS (PE Ch iCS, black) or SE Ch (SE Ch, magenta) Env proteins and imaged by 3D spinning-disk confocal microscopy at different time points after warming the cells to 37°C. *Z*-stacks with 30 planes were collected every 5 to 10 min. The data points at 0 min represent the colocalization percentage obtained from the corresponding sample preparations directly spotted on a cover slide (as shown, for example, in Figure 1D). The colocalization percentage obtained from several HeLa cells for each of the three virus strains is shown as a function of time. The colocalization percentage was determined by grouping the measurements into subsets of 2–4 cells and calculating the average percentage (circle) of colocalization and the standard deviation of the mean (error bars) (PE Ch: N = 13, 5 subsets; PE Ch iCS: N = 16, 5 subsets; SE Ch: N2009= 23, 7 subsets). The results were averaged into 10-minute time bins. Lines are drawn between data points as a guide to the eye

HeLa cells were incubated with virus particles at ~10°C, and the temperature was then shifted to 37°C to allow viral uptake and/or fusion. From the 3D colocalization analysis, we determined the total number of Gag-eGFP particles and the number of double-tagged virus particles in individual cells over time. We avoided high virus concentrations to ensure more virological relevant conditions and to minimize the possibility of random colocalization of capsid and Env signal. The typical number of virions per cell varied between 50 and 100 for these experiments, although up to 180 Gag-eGFP particles could be analyzed without difficulty as the analysis was performed in 3D and the number of particles detected within one z-plane was low enough to allow a reliable colocalization analysis (Figure 9, see Additional file 7). In order to estimate the uncertainty of the measurement, the data were divided into 5–7 subsets gathered from 2–4 cells and binned in 10 minute intervals starting from when the temperature reached 37°C. We analyzed the colocalization percentage over time for the PFV Gag-eGFP-labeled virions containing three different envelope proteins: PE Ch, PE Ch iCS and SE Ch (Figure 9). The PE Ch iCS particles possess no significant fusion activity and served as a control. The measured colocalization percentage for these particles remained constant around 96% throughout the measurement time of 90 minutes (Figure 9, black data points). This value corresponds to the colocalization percentage for the same batch of double-labeled viruses (95 ± 1%) measured after spotting on cover slides (Figure 3D). SE Ch virus preparations showed a colocalization percentage of 93% and in live-cell measurements a slow decay was observed over time from 92 ± 2% in the first 20 minutes to around 85 ± 2% after 1.5 h pb (Figure 9, magenta data points). In the case of PE Ch particles, a significant drop in the colocalization percentage was already observed at the first time point (Figure 9, blue data points). A colocalization percentage of 93 ± 1% was determined for the virus preparation on coverslips and dropped below 80% in the first 30 minutes in the live-cell measurements. This drop was significantly below the colocalization percentage determined for both SFVmac and the non-fusogenic control PE Ch iCS.

The percentage of colocalizing particles was always determined with respect to the number of detected Gag-eGFP particles. This approach minimizes artifacts arising from quenching of the eGFP signal at low pH. When the eGFP signal of the double-tagged virus particles is quenched, the particle will not be included in the analysis. In addition, quenching of the FP signal was not a significant issue for this sample. We measured the effect of low pH on our virus particles and only a slow decay over several minutes of the Gag-eGFP signal at pH 5.5 was observed (data not shown). No quenching of the Env-labeled mCherry was observed at low pH (data not shown).

A slight increase in the colocalization percentage of PE Ch particles was observable between 30 to 50 minutes pb. Although the increase is near the limit of statistical relevance, formation of large aggregates of both Gag and Env signal in the perinuclear region made it impossible to analyze individual particles in this region. Both the limited sensitivity in the perinuclear region as well as capsid disassembly would lead to a decrease in the detection of green only particles (Figure 5) and thus to an increase in the colocalization percentage. The second decay, starting after about 50 minutes, may result from a different entry pathway that becomes more prominent at this time point, e.g. fusion with (late) endosomes in contrast to fusion at the plasma membrane. However, this is currently speculation.

In summary, the observed colocalization percentage for PE Ch and SE Ch particles was always below the non-fusogenic control sample PE Ch iCS. For PE Ch particles, a significant drop in the colocalization percentage was observed within the first 10 minutes, whereas for SE Ch particles, the decay was slower and occurred throughout the length of the measurement. The slower kinetics observed for SE Ch particles can be, at least in part, attributed to the requirement of endosomal acidification to trigger the fusion process. In contrast, PFV particles were shown to already possess a significant fusion activity at neutral pH [16], which is consistent with our observation of early fusion events. Additionally, these findings are in agreement with the observation of syncytia formation, which was only observed for cells incubated with PE Ch particles (Figure 4A). Based on these findings, the timescale to expect most fusion events would be expected to occur during the first 30 min post attachment.

## Conclusions

The generated FVs with FP-tagged capsids and glycoproteins provide an excellent tool for investigating the early steps of viral entry. We demonstrated that, in individual living cells, the attachment and uptake of viral like particles is independent of the fusion activity of the viral glycoprotein. The majority of fusion events appear to occur within the first two hours post entry. Virions that haven’t released their capsids into the cytosol within the first six hours are prone for degradation. In line with previous reports [18,20], capsids released into the cytoplasm accumulate at the MTOC, and Gag proteins gain access to the cellular genome upon mitosis. Our results suggest that there are differences in the uptake pathways of various FV species determined predominantly by the type of FV glycoprotein utilized. PFV Env containing virions release their capsids, to a large extent, within the first few minutes after binding. This suggests that PFV can fuse with the plasma membrane, which is supported by the fusogenic activity of the Env protein at neutral pH and the high cell-cell fusion activity observed. In contrast, SFVmac Env containing viruses appear to require endocytosis and acidification for fusion to occur. Thus, the double-FP-tagged FVs introduced in this study provide a very powerful tool for detailed analyses of the early steps of FV entry and promise to be useful for visualizing the fusion process itself.

## Methods

### Cells

The human kidney cell line 293T [45], the human fibrosarcoma cell line HT1080 [46] and the human cervical HeLa cell line [47] were cultivated in Dulbecco’s modified Eagle’s medium supplemented with 10% heat-inactivated fetal bovine serum (FBS) and antibiotics. During live-cell imaging assays, HeLa cells were kept in Leibovitz’s L15 medium, supplemented with 10% FBS.

### Expression constructs

The 4-component PFV vector system consisting of the PFV Gag expression vector pcoPG4, the PFV Pol expression vector pcoPP, the PFV Env expression construct pcoPE (PE), and the enhanced green fluorescent protein (eGFP)-expressing PFV transfer vector puc2MD9 or pMD11, encoding lacZ as a reporter gene, has been described previously [13,48,49]. In some experiments, the corresponding not codon-optimized expression constructs pcziGag4, pcziPol and pciSE (SE) [16,48] were used. Expression vectors for FV mCherry-Env fusion proteins were cloned by fusing the FP tag sequence connected by a flexible glycine-serine (G/S) linker to the N-terminus of PFV Env in pcoPE (pcoPE Ch) or SFVmac Env in pciSE (pciSE Ch) (see Figure 2). Further modification of these Env expression constructs by insertion of the amino acid exchanges R_571_T in PFV Env [34] or RKRR_570-573_AAEA in SFVmac Env [50] resulted in the generation of the corresponding SU-TM cleavage site mutants (pcoPE Ch iCS, pciSE Ch iCS). All FP-tagged expression constructs were generated using standard PCR cloning techniques and mutagenesis primers and were verified by sequencing analysis. Details of the cloning procedure and primer sequences are available upon request.

The pgTubulin-DsRed expression plasmid encoding a gamma-tubulin-dsRed fusion protein was obtained from Euroscarf [51].

### Virus production

Recombinant PFV particles and HIV pseudoparticles were essentially produced and harvested from polyethylenimine (PEI) transfected cells as described previously [13,52,53]. Briefly, PFV containing supernatants were generated by cotransfection of 293T cells with transfer vector puc2MD9, Pol- (pcoPP), Env- (pcoPE, pcoPE Ch, pcoPE Ch iCS) and Gag packaging plasmid (pcoPG4, pcoPG4 CeGFP) at a ratio of 28:2:1:4. SFVmac containing supernatants were produced by cotransfection of puc2MD9, pcziPol, pcziGag4 (or pcziGag-CeGFP) and Env packaging plasmids (pciSE, pciSE Ch, pciSE Ch iCS) at a ratio of 1:1:1:1. For live-cell imaging experiments, the transfer vector pMD11 instead of puc2MD9 and the enzymatic inactive reverse transcriptase encoding Pol packaging plasmid pcoPP2 [49] instead of pcoPP were used. At 24 h post-transfection, sodium butyrate (final concentration, 10 mM) was added to the growth medium. At 8 h post induction, the cell culture medium was replaced and, after an additional 16 h, viral supernatants were harvested.

### Analysis of transduction efficiency

Transduction of host cells by HIV pseudoparticles, PFV or SFVmac Env containing viral supernatants was performed by infection of 2 x 10^4^ HT1080 cells, plated 24 h in advance in 12-well plates. During the incubation period (4–6 h), target cells were covered with 1 ml of the viral supernatant or dilutions thereof prior to media replacement. The percentage of eGFP-positive cells was determined by flow cytometry analysis 72 h after infection. All transduction experiments were performed three times and, in each independent experiment, the titers obtained with the untagged wild-type viruses were arbitrarily set to 100% and those of the other samples expressed as values relative to the wt control, as described previously [54].

In some experiments, target cells (HeLa) were incubated at different concentrations with Bafilomycin A1 (Sigma-Aldrich), Chloroquin-Diphosphat (AppliChem), Genistein (Sigma-Aldrich), Nystatin (Merck), Dynasore (Sigma-Aldrich). After a 1 h preincubation period at 37°C, HeLa cells were exposed to PFV or SFVmac Env containing FVs or VSV-G enveloped HIV pseudoparticles for 4 h in the presence of drugs. The supernatant was substituted with fresh drug-containing cell culture medium for an additional hour before cultivation in medium without drug. Seventy-two hours later, the percentages of EGFP-expressing HeLa cells were determined by flow cytometry.

### Viral particle purification for immunoblotting

Ten milliliters of cell-free viral supernatant were harvested by sterile filtration (pore size, 0.45 μm) and centrifuged at 4°C and 25,000 rpm for 3 h in an SW40 (Beckman) rotor through a 20% sucrose cushion. Subsequent to centrifugation, the supernatant was discarded and the viral pellet was resuspended in 100 μl 1xPPPC (sodium dodecyl sulfate (SDS) protein sample buffer).

### Cell lysates, antisera, immunoblotting

A transfected 10 cm dish was prepared for cell lysis by incubation with 600 μl lysis buffer (10 mM Tris–HCl (pH 8), 140 mM NaCl, 0,025% NaN3, 1% TritonX-100) and subsequently centrifugation through QIAshredder (Qiagen) columns. Cell lysates and purified particles were separated by SDS-polyacrylamide gel electrophoresis (PAGE) and analyzed by immunoblotting. The polyclonal antisera used were specific for PFV Gag [55], the LP of PFV Env (aa 1 to 86) [22], the LP of SFVmac Env (aa 2–69) or mCherry (399C). The monoclonal antisera used were raised against PFV SU (P3E10) [56] or eGFP (Roche). In some experiments, specific antibodies raised against the cellular housekeeping protein GAPDH (Glyceraldehyde 3-phosphate dehydrogenase) were used (Sigma). The chemiluminescence signal was digitally recorded using a LAS-3000 imager.

### Production of antisera

SFVmac LP specific antiserum (SFVmac Env (aa 2–69)) was generated by insertion of a PCR fragment encoding SFVmac Env aa2-69 in-frame downstream of the maltose binding protein (MBP) ORF of the prokaryotic pMAL-C2 expression vector (New England Biolabs). The mCherry-specific antiserum (399C) was produced by insertion of a mCherry-decaHis fusion protein ORF (His, Histidine) into the prokaryotic pET11 expression vector (Novagen). The soluble fusion proteins were expressed in *Escherichia coli* TB1 or BL21(DE3) cultures after induction with 0.5 mM isopropylthiogalactopyranoside (IPTG) for 3 to 6 h respectively and affinity purified according to the manufacturer’s instructions.

### Analysis of viral proteins in infected cells

HeLa cells were seeded at a density of 5 x 10^4^ cells/well in 12 well plates one day prior to the experiments. If the assay was performed in the presence of Bafilomycin A1 (BAF), the target cells were preincubated with BAF (60 nM) for 1 h at 37°C. After pre-cooling to 12°C, the cells were incubated with 1 ml virus-containing cell culture media (+/− BAF (60 nM)), that had been harvested and concentrated (20x) by low speed centrifugation (14,000g, 1.5 h, 4°C). Following 30 minutes incubation at 12°C, the cells were either rinsed with PBS and prepared for cell lysis (0 h pb) or the media was replaced by fresh 10% DMEM (+/− BAF (60 nM)) prior to warming the cells to 37°C (1–24 h pb). At the given time points after the cell had reached 37°C, the cells were rinsed with PBS and incubated with 2xPPPC followed by centrifugation through QIAshredder (QIAgen) columns. Cell lysates were separated by SDS-polyacrylamide gel electrophoresis (PAGE) and analyzed by immunoblotting.

### Purification and concentration of FV particles for imaging analysis

Fluorescent FV particles were produced as described above using the pMD11 transfer vector. Subsequent to ultracentrifugation (25,000 rpm) of 30 ml cell culture supernatant in a SW28 rotor (Beckman), the viral pellet was gently resuspended in 90 μl PBS supplemented with 10% FBS resulting in a 333x volume concentration. In some experiments, viral particles were harvested by using Pierce® concentrators (150K, Thermo Scientific). In that case, the cell culture media of transfected 293T cells was substituted with phenol red- and FBS-free DMEM after sodium-butyrate induction. With this method, an equal concentration factor was obtained. The viral particles were stored as aliquots at −80°C.

### Wide-field live-cell microscopy

HeLa cells were seeded at a density of 0.5 x 10^4^ cells/200 μl into one well of an eight-chamber slide (IBIDI, cat. No: 80826). The cell culture media was replaced 24 h later by cold L15 media supplemented with 10% FBS (+/− BAF (60 nM)) and the cells were cooled to 10°C (5 min). Afterwards, 3–5 μl of purified fluorescent FV particle preparations were added for 30 minutes at 10°C. Subsequently, cells were washed twice with cold L15 media (+/− BAF (60 nM)). Cells were then warmed to 37°C, and live-cell microscopy was started immediately. A z-stack was collected every 15 minutes using 600 nm spacing between consecutive planes and 10 to 12 planes total. Wide-field images were collected on a Nikon TE2000E using a Nikon Plan Fluor 40x (numerical aperture 1.3) oil immersion objective. The light of a mercury lamp was used to alternately excite eGFP (BP470/30) and mCherry (BP590/20). The emission signals were passed through a dichroic mirror and a 465/50 or 545/60 bandpass filter prior to detection with a CCD camera. The movies and images were evaluated with ImageJ (http://rsb.info.nih.gov/ij/).

### Spinning-disk confocal microscopy

One day prior to the measurements, HeLa cells were seeded at a density of 2 x 10^4^ cells/400 μl in one well of an eight-chamber slide (Lab-Tek). Prior to virus incubation, the cell-culture medium was replaced by L15 medium and the cells were cooled to ~10°C (10 min). 1–3 μl of purified virus were added in the vicinity of the HeLa cells to be measured and allowed to bind for an additional 10 minutes at ~10°C. Subsequently, the cells were rinsed with cold L15 medium and the imaging was started immediately after warming the cells to 37°C. The spinning-disk confocal microscope system (Revolution System; Andor Technology) utilized a Nikon microscope base (TE2000E) and the spinning-disk unit CSU10 from Yokogawa. Measurements were performed with an oil immersion total internal reflection fluorescence (TIRF) objective (60x, NA = 1.49, Nikon) in combination with a 1.5x tube lens. The detection path was equipped with an Optosplit II (Cairn Research Ltd.) for dual-color detection, a filter set for eGFP and mCherry (BS562, HC525/50 and ET605/70; AHF Analysentechnik AG) and a DU-897 Ixon EMCCD camera (Andor). In addition, a triple-band dichroic beam splitter was used to separate laser excitation from fluorescence emission (Di01-T405/488/568/647; Semrock). The excitation was controlled with an acousto-optic tunable filter (AOTF). The sample position was controlled with an *xyz* piezo stage (ProScan II, NanoScanZ; Prior Scientific). Multi-fluorescent beads (TetraSpeck microspheres, 0.1 μm, Invitrogen) immobilized on a coverslip were used to calibrate the overlap of the two detection channels. Multiple cells were measured sequentially during one experiment by recording the *xy* positions of several cells and automatically moving the *xy* stage to the appropriate positions during the experiment. The corresponding time interval between *z*-stacks for each cell was varied between 5 and 10 minutes.

### 2D colocalization analysis

An analysis program was developed in house to determine the amount of colocalization within a 2D image based on an intensity ratio of the particles. Particles in each channel were fitted to a 2D Gaussian and selected by the criteria of particle size, intensity threshold and minimal distance between two neighboring particles. Colocalization was determined based on an intensity ratio of the particles detected in the green channel with respect to the intensity in the red channel. Particles with a ratio around one were defined as colocalized.

### 3D colocalization analysis

For the 3D colocalization analysis, a software program was developed in house to determine the amount of colocalization from a *z*-stack of images based upon the minimum distance between particles detected in the green and red channels. Particles were initially detected using a spot-enhancing filter and an intensity threshold within the three-dimensional image volume. The position of each particle was estimated by calculating the center of mass of the fluorescence intensity for each spot. The image plane containing the highest intensity coming from the particle was taken and the lateral position of the particle determined by fitting the intensity to a 2D Gaussian function. The axial-position was taken as the position of the *z*-plane with the maximum intensity, which was accurate to within ±150 nm. The green and red channels were recorded alternatively. As autofluorescence has a very broad excitation spectrum, fluorescence structures that had a strong signal in the red channel after 488 nm excitation were assigned as cellular autofluorescence and excluded from the analysis to avoid false positive colocalizations. Particles were detected independently in the green and red channel for each z-plane and z-stack based on an intensity threshold, particle size criteria and a minimal distance between two neighboring particles. As the two channels were recorded alternatively with ~150 ms delay, the positions of the green and the red signals could differ slightly due to motion of the dual-color particle. This shift in position was taken into account by calculating the distance between the signals detected in the two channels and introducing a maximally allowed displacement of 2.2 μm in the *xy* plane and 600 nm between the *z*-planes. We chose the separation tolerance to be relatively high to ensure that colocalizing particles that are undergoing transport are identified as colocalizing particles and do not yield false positive fusion results. Thus, a detectible decrease in colocalization percentage would clearly imply that fusion is occurring. Colocalization of particles in different channels was based on the three-dimensional separation of the particles.

### Confocal laser scanning microscopy

HeLa cells were seeded at a density of 2 x 10^4^ cells/ml into 12-Well plates on glass cover slips. After 24 h, the cells were transfected with 0.5 μg of the pgTubulin-DsRed expression plasmid using FuGENE® HD transfection reagent according to the manufacturers instructions. Another 24 h later, the transfected cells were precooled and incubated on ice with Gag-eGFP fluorescent SFVmac particle preparations for 30 minutes. Subsequently, the cells were washed with cold PBS and either fixed with 3% PFA or incubated an additional 2 or 6 h at 37°C prior to fixation. Following DAPI staining, the samples were covered in Mowiol. Confocal laser scanning images were obtained on a Zeiss LSM 510 as described previously and evaluated by ImageJ [13].

### Electron microscopy analysis

HeLa cells were seeded at a density of 1x10^6^ cells/well in 6 well plates one day prior to measuring. After precooling, the cells were incubated with untagged wildtype PFV particles (MOI 10) produced as described above using the 4-component vector system. After 30 min incubation, the cells were either fixed or shifted to 37°C for an additional 10 or 30 min prior to fixation. The cells were harvested and processed for electron microscopy analysis as described previously [57].

## Competing interests

The authors declare that they have no competing interests.

## Authors’ contributions

DL and DCL conceived and coordinated the study. KS, DS, AD, EM, VK and JR performed the experiments or generated essential materials used in the study. KS, DS, AD, DL and DCL were involved in data interpretation and drafting of the manuscript. All authors read and approved the final manuscript.

## Supplementary Material

Additional file 1 **Movie.** Comparison of syncytia formation in PFV or SFVmac infected cells. HeLa cells were incubated with double-tagged PFV Gag-eGFP (non-spiked) and either PE Ch or SE Ch containing viral particles at low temperature (~10°C). After warming the cells to 37°C, *z*-stacks with 13 planes were recorded every 15 minutes for a total of 7.5 h using wide-field live-cell microscopy. In the upper part of the video, the DIC images for the PE Ch containing particles (left panels) or SE Ch containing particles (right panels) are shown. In the lower part of the movie, the corresponding fluorescence overlay images of the red (Ch-Env) and the green (Gag-eGFP) channels are depicted. The overlay images are displayed as maximum intensity *z*-projection of each *z*-stack. The DIC channel was adjusted according to brightness and contrast using ImageJ PlugIns. Scale bar: 20 μm. Click here for file

Additional file 2 **Figure.** Influence of various drugs on host cell transduction by retroviral vectors pseudotyped with different glycoproteins. HeLa cells were preincubated with the appropriate drugs (1 h, 37°C) in two different concentrations (as indicated) prior to exposure to PFV or SFVmac Env containing FVs or VSV-G enveloped HIV pseudoparticles for 4 h in the presence of drugs. The supernatant was substituted with fresh drug-containing cell culture medium for an additional hour before cultivation in medium without drug. Seventy-two hours later, the percentages of EGFP-expressing HeLa cells were determined by flow cytometry. Relative infectivities obtained without drug-treatment (i.e. medium only), typically 30-40% eGFP-positive cells, were set arbitrarily to 100%. Displayed are the mean and standard deviation of two independent experiments. BAF (Bafilomycin A1), CHL (Chloroquine), DYN (Dynasore), NYS (Nystatin), and GEN (Genistein). Click here for file

Additional file 3 **Movie.** Uptake and trafficking of incoming wild-type PFV particles in host cells. HeLa cells were incubated with double-tagged PFV Gag-eGFP (non-spiked) and PE Ch containing viral particles at low temperature (~10°C). After warming the cell to 37°C, *z*-stacks with 10 planes were recorded every 15 minutes for a total of 24 h using wide-field live-cell microscopy. The video shows the DIC channel (upper left), the red Ch-Env channel (upper right) and the green Gag-eGFP channel (lower left), both shown in gray, and a colored overlay of both channels with Gag-eGFP in green and Ch-Env in red (“merge”, lower right). The individual fluorescence channels and the “merge” channel are displayed as maximum intensity *z*-projection of each *z*-stack. The DIC channel was adjusted according to brightness and contrast using ImageJ PlugIns. Click here for file

Additional file 4 **Movie.** Gag tethering to host cell chromatin during mitosis. HeLa cells were incubated with double-tagged PFV Gag-eGFP (non-spiked) and either PE Ch or PE Ch iCS containing viral particles at low temperature (~10°C). After warming the cells to 37°C, *z*-stacks with 12 planes were recorded every 15 minutes for a total of 6 h using wide-field live-cell microscopy. The video shows the DIC channel (upper panels), the Gag-eGFP channel (middle panels) displayed in gray and an overlay of these two channels (lower panels) for the PE Ch containing particles (right channels) and PE Ch iCS containing particles (right channels). The individual fluorescence channels and the “merge” channel are displayed as maximum intensity *z*-projection of each *z*-stack. The DIC channel was adjusted according to brightness and contrast using ImageJ PlugIns. Click here for file

Additional file 5 **Movie.** Uptake and trafficking of fusion-deficient PFV particles in host cells. HeLa cells were incubated with double-tagged PFV Gag-eGFP (non-spiked) and PE Ch iCS containing viral particles at low temperature (~10°C). After warming the cell to 37°C, *z*-stacks with 12 planes were recorded every 15 minutes for a total of 24 h using wide-field live-cell microscopy. The video shows the DIC channel (upper left), the red Ch-Env channel (upper right) and the green Gag-eGFP channel (lower left), both shown in gray, and a colored overlay of both channels with Gag-eGFP in green and Ch-Env in red (“merge”, lower right). The individual fluorescence channels and the “merge” channel are displayed as maximum intensity *z*-projection of each *z*-stack. Click here for file

Additional file 6 **Movie.** FV uptake and trafficking in Bafilomycin A1 treated cells. Hela cells pretreated with 60 nm BAF were incubated with double-tagged PFV Gag-eGFP (non-spiked) and either PE Ch, SE Ch or SE Ch iCS containing viral particles at low temperature (~10Â°C) in the presence of the drug. After warming the cell to 37°C, *z*-stacks with 13 planes were recorded every 15 minutes for a total of 12 h using live-cell wide-field microscopy. The video shows the DIC channel (upper channels), the green Gag-eGFP channel (middle channels) shown in gray and a colored overlay of both channels with Gag-eGFP in green and Ch-Env in red (“merge”, lower channels). The individual fluorescence channels and the “merge” channel are displayed as maximum intensity *z*-projection of each *z*-stack. scale bar: 10 μm. Click here for file

Additional file 7 **Figure.** HeLa cell with representative particle number during confocal time-lapsed analysis of colocalized capsid and Env signal in live cells. HeLa cells were incubated with PFV PE-Ch at ~ 10°C for 10–15 min, warmed to 37°C and imaged for 90 minutes. One *z*-slice from a representative cell containing a total of ~50 particles is shown 22 min after reaching 37°C. (A) The signal from the Gag-eGFP channel, (B) the mCherry Env channel and (C) a merged image are shown. The circle indicates the allowed displacement parameter in *x* and *y* of 2.2 μm around the center of each Gag-eGFP particle. When a red particle is detected within this area and is within 600 nm in z (dashed circle in panel (B)), particles are defined as colocalizing (yellow circle in panel (C)). When no red particles are detected within this area, the green particle is defined as non-colocalizing (green circle in panel (C)). (D) A bright-field image of the corresponding HeLa cell at the end of the experiment. The approximate boarders of the membrane are indicated by a white line. Scale bar 10 μm. Click here for file
