# Supplementary figures and images for: Differential pH-dependent cellular uptake pathways among foamy viruses elucidated using dual-colored fluorescent particles
Source: Retrovirology. 2012 Aug 30;9:71. doi: 10.1186/1742-4690-9-71 (PMC3495412; doi:10.1186/1742-4690-9-71)

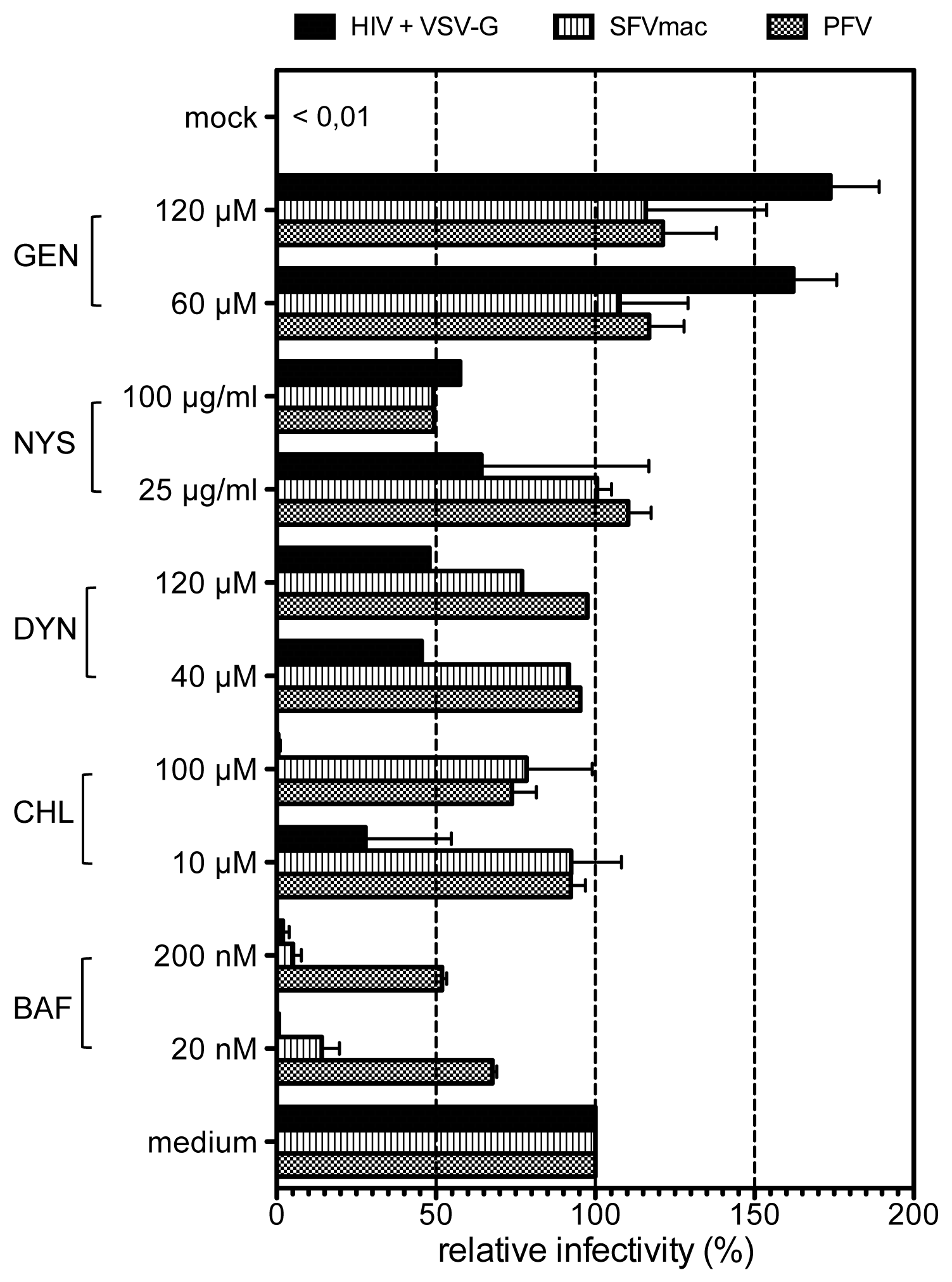

Supplement: Additional file 2 — Figure. Influence of various drugs on host cell transduction by retroviral vectors pseudotyped with different glycoproteins. HeLa cells were preincubated with the appropriate drugs (1 h, 37°C) in two different concentrations (as indicated) prior to exposure to PFV or SFVmac Env containing FVs or VSV-G enveloped HIV pseudoparticles for 4 h in the presence of drugs. The supernatant was substituted with fresh drug-containing cell culture medium for an additional hour before cultivation in medium without drug. Seventy-two hours later, the percentages of EGFP-expressing HeLa cells were determined by flow cytometry. Relative infectivities obtained without drug-treatment (i.e. medium only), typically 30-40% eGFP-positive cells, were set arbitrarily to 100%. Displayed are the mean and standard deviation of two independent experiments. BAF (Bafilomycin A1), CHL (Chloroquine), DYN (Dynasore), NYS (Nystatin), and GEN (Genistein). [file 1742-4690-9-71-S2.tiff]

Gag-eGFP

mCherry-Env

overlay

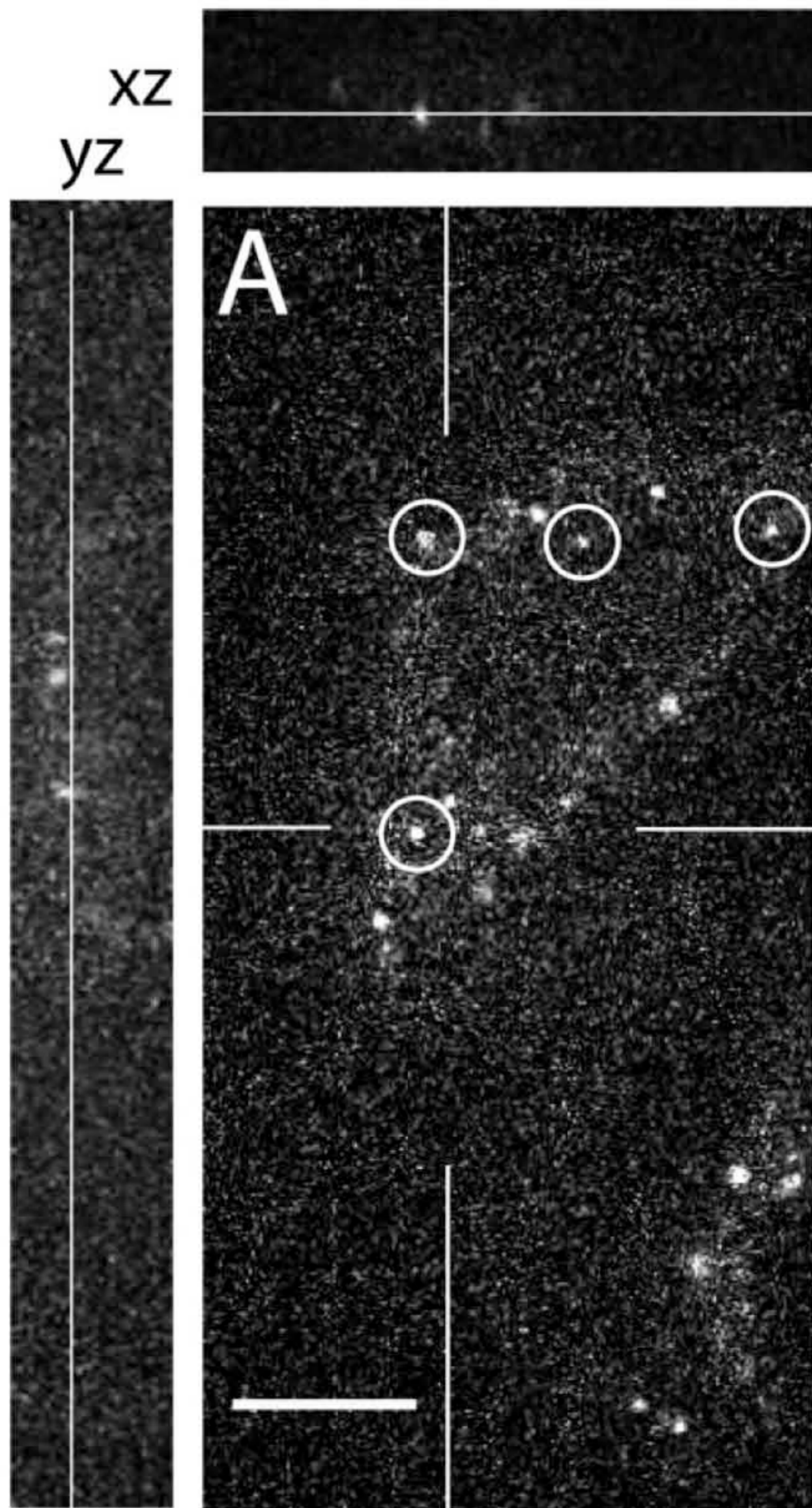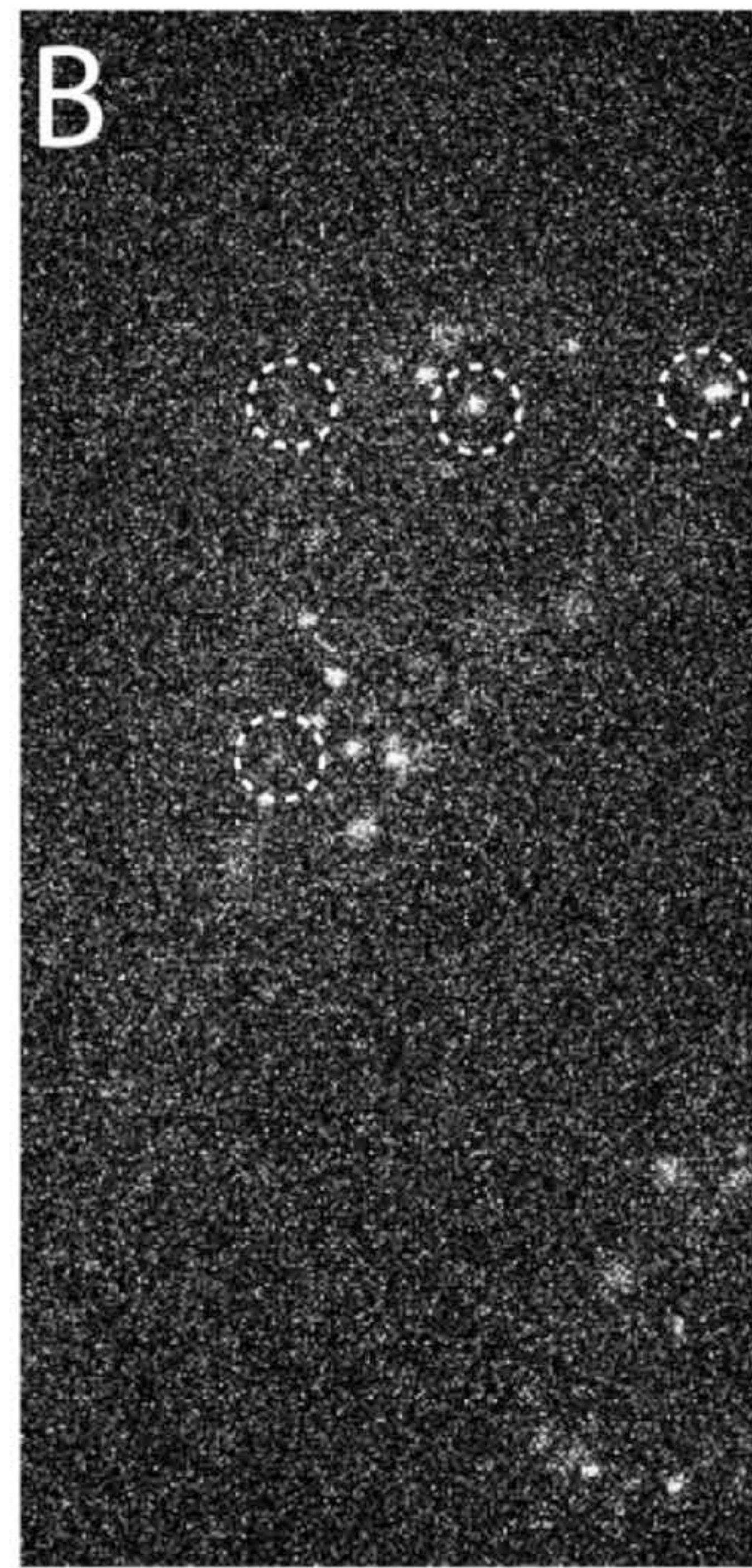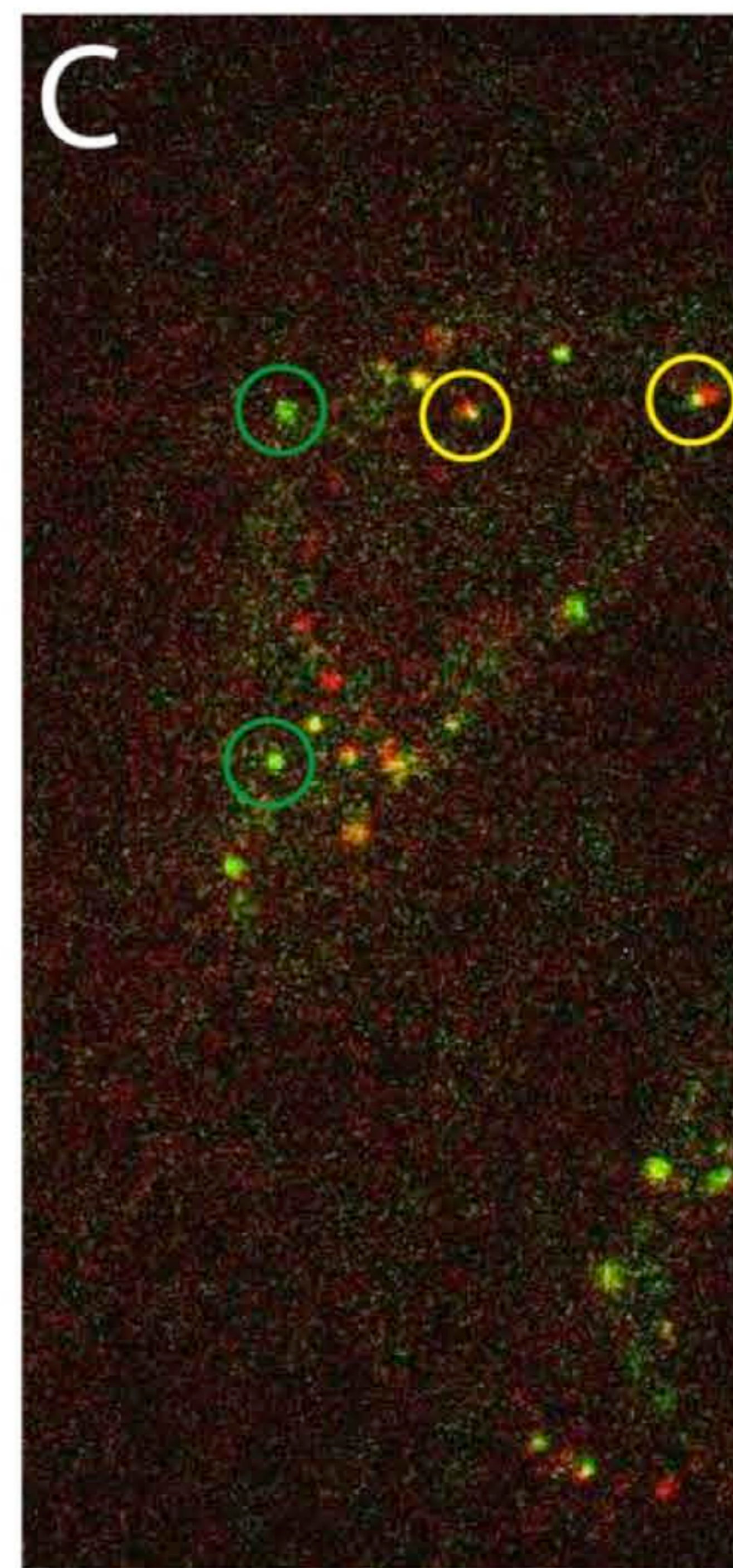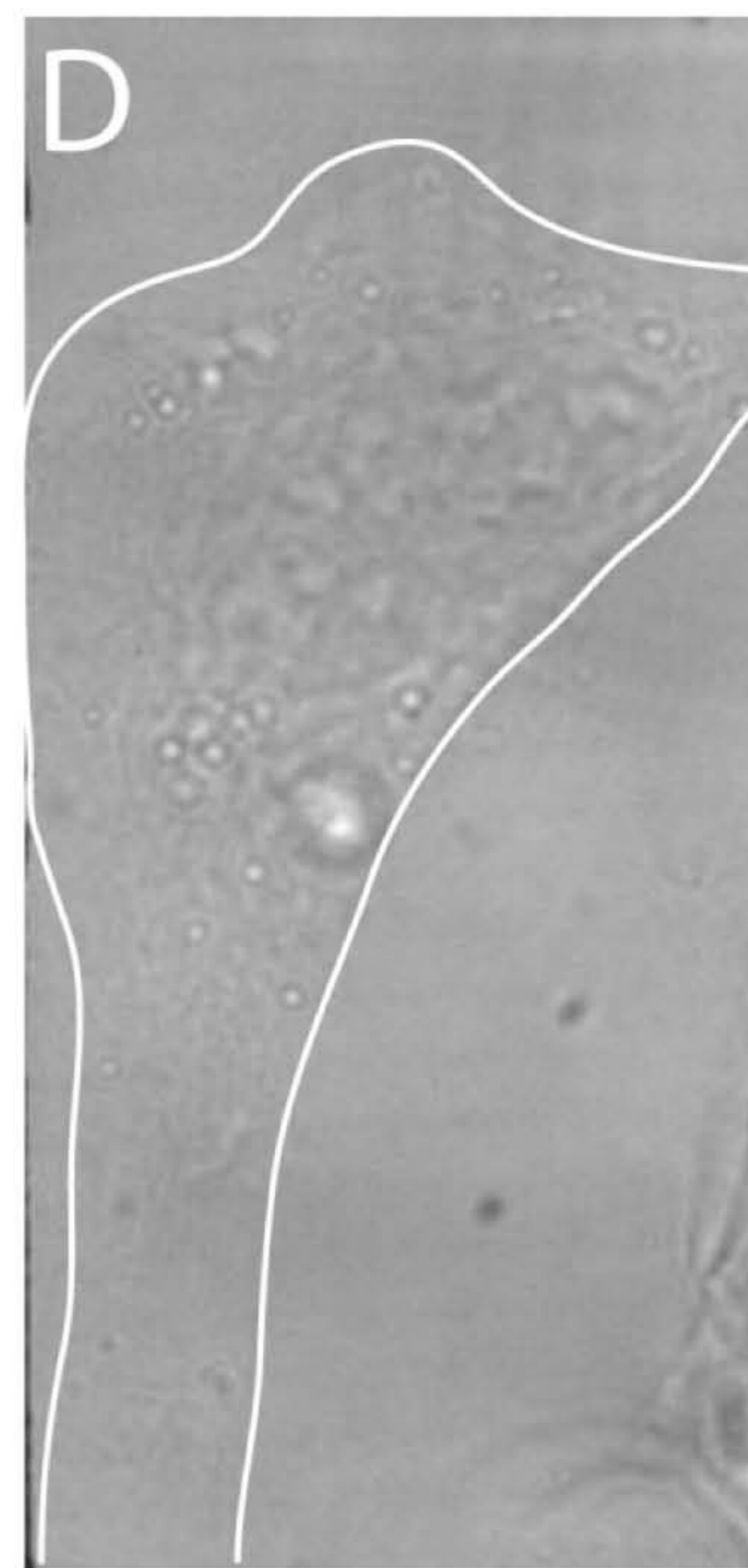

Supplement: Additional file 7 — Figure. HeLa cell with representative particle number during confocal time-lapsed analysis of colocalized capsid and Env signal in live cells. HeLa cells were incubated with PFV PE-Ch at ~ 10°C for 10–15 min, warmed to 37°C and imaged for 90 minutes. One z-slice from a representative cell containing a total of ~50 particles is shown 22 min after reaching 37°C. (A) The signal from the Gag-eGFP channel, (B) the mCherry Env channel and (C) a merged image are shown. The circle indicates the allowed displacement parameter in x and y of 2.2 μm around the center of each Gag-eGFP particle. When a red particle is detected within this area and is within 600 nm in z (dashed circle in panel (B)), particles are defined as colocalizing (yellow circle in panel (C)). When no red particles are detected within this area, the green particle is defined as non-colocalizing (green circle in panel (C)). (D) A bright-field image of the corresponding HeLa cell at the end of the experiment. The approximate boarders of the membrane are indicated by a white line. Scale bar 10 μm. [file 1742-4690-9-71-S7.pdf]
